# Inducing cellular senescence *in vitro* by using genetically encoded photosensitizers

**DOI:** 10.18632/aging.101065

**Published:** 2016-10-14

**Authors:** Nadezhda V. Petrova, Artem V. Luzhin, Ekaterina O. Serebrovskaya, Alina P. Ryumina, Artem K. Velichko, Sergey V. Razin, Omar L. Kantidze

**Affiliations:** ^1^ Institute of Gene Biology, Russian Academy of Sciences, 119334 Moscow, Russia; ^2^ Shemyakin-Ovchinnikov Institute of Bioorganic Chemistry, Russian Academy of Sciences, 117997 Moscow, Russia; ^3^ Department of Molecular Biology, Lomonosov Moscow State University, 119991 Moscow, Russia; ^4^ LIA 1066 French-Russian Joint Cancer Research Laboratory, 94805 Villejuif, France

**Keywords:** KillerRed, miniSOG, DNA damage, optogenetics, cellular senescence

## Abstract

Cellular senescence, a form of cell cycle arrest, is one of the cellular responses to different types of exogenous and endogenous damage. The senescence phenotype can be induced *in vitro* by oncogene overexpression and/or DNA damage. Recently, we have reported a novel mechanism of cellular senescence induction by mild genotoxic stress. Specifically, we have shown that the formation of a small number of DNA lesions in normal and cancer cells during S phase leads to cellular senescence-like arrest within the same cell cycle. Here, based on this mechanism, we suggest an approach to remotely induce premature senescence in human cell cultures using short-term light irradiation. We used the genetically encoded photosensitizers, tandem KillerRed and miniSOG, targeted to chromatin by fusion to core histone H2B to induce moderate levels of DNA damage by light in S phase cells. We showed that the cells that express the H2B-fused photosensitizers acquire a senescence phenotype upon illumination with the appropriate light source. Furthermore, we demonstrated that both chromatin-targeted tandem KillerRed (produces O_2_^−^) and miniSOG (produces ^1^O_2_) induce single-stranded DNA breaks upon light illumination. Interestingly, miniSOG was also able to induce double-stranded DNA breaks.

## INTRODUCTION

Cellular senescence is growth arrest characterized by complex phenotypic changes and loss of re-proliferative potential [1, 2]. To study cellular senescence, one can transfect cells with a plasmid that encodes a constitutively active oncogene, thus inducing the so-called oncogene-induced senescence (OIS) [3, 4]. It is thought that in course of OIS cells, which are cell cycle-arrested due to oncogene-induced DNA damage, undergo geroconversion stimulated by activation of mTOR pathway [1, 5]. Mechanistically, oncogene-induced DNA damage may result from DNA hyper-replication [6], replication fork reversal [7], the depletion of nucleotide pools [8] and/or increased levels of reactive oxygen species (ROS) [9]. OIS can be stimulated in a DNA damage response (DDR)-independent manner as well, specifically through the activation of CDKN2A genomic loci that code for p16^INK4a^ and ARF [10, 11]. Obviously, the expression of activated oncogenes induces an excessively complex composition of senescence-inducing stimuli, making OIS a difficult-to-interpret model [4, 12]. Cellular senescence can be induced by sublethal concentrations of DNA-damaging agents as well; however, in this case, an extremely long incubation time (from hours to days) is usually needed [13]. It is also worth noting that low molecular weight compounds may act in many (sometimes unexpected) ways. For instance, a topo-isomerase II inhibitor, doxorubicin, which is known to induce premature senescence, stimulates ROS overproduction and histone eviction, as well as topoisomerase-dependent DNA damage [14–16]. Thus, it is reasonable to develop a simple and versatile method to induce cellular senescence in normal and cancer cell lines.

Here, we propose a novel approach to induce cellular senescence *in vitro* using the genetically encoded photosensitizers tandem KillerRed (tKR, a modified version of KillerRed [17, 18]) and miniSOG (mini singlet oxygen generator) [19, 20]). It is generally thought that upon illumination, photosensitizers produce ROS by a Type I (superoxide anion radicals, hydrogen peroxides and hydroxyl radicals) or Type II (singlet oxygen) photosensitization reaction [21]. Irradiation of the photosensitizers can induce different types of cell death (apoptosis, necrosis, or autophagy), depending on the cellular compartment to which they are targeted [21]. There are only a few genetically encoded photo-sensitizers [18, 19, 22]; among them, KR is the first developed and the best-studied one, and miniSOG is the most promising, due to its enhanced efficiency of ROS production and relatively low molecular weight [23]. It was proven that KR activation results in superoxide anion radical production (O_2_^−^), while miniSOG activation results in singlet oxygen production (^1^O_2_) [23–26]. The cell killing induced by photosensitizers usually depends on their ROS-mediated proteotoxic effects [21]. However, it has recently been shown that KR activation in the cell nucleus can lead to DNA damage and may be used for light-induced inhibition of cell division [17] and for localized induction of oxidative DNA lesions [27]. Nevertheless, it is still debatable whether O_2_^−^ and ^1^O_2_ can induce single- and/or double-stranded DNA breaks (SSBs and DSBs), as well as the oxidation of DNA bases [28, 29]. In the present study, we analyzed the DNA-damaging effects of tKR and miniSOG, which were targeted to the cell nucleus by fusing them to the core histone H2B. We found that although both photosensitizers effectively stimulate SSB formation upon light irradiation, miniSOG can additionally induce a small number of DSBs. Based on our recently reported mechanism of mild genotoxic stress-dependent cellular senescence [30], we employed these genetically encoded photo-sensitizers to induce a cellular senescence-like state.

## RESULTS AND DISCUSSION

### Overview of the approach

**Figure 1:**
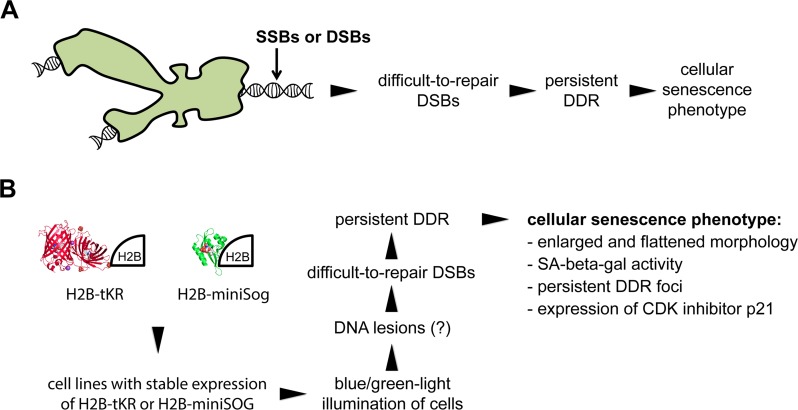
Overview of the method used to optogenetically induce cellular senescence in vitro (**A**) Model illustrating how mild genotoxic stress can induce cellular senescence-like proliferation arrest (according to [30]). (**B**) Overview of the method for inducing cellular senescence using the genetically encoded photosensitizers tandem KillerRed (tKR) and miniSOG that were targeted to chromatin.

Recently, we reported a novel mechanism of cellular senescence induction by mild genotoxic stress [30, 31]. Specifically, we showed that formation of a small number of DNA lesions in normal and cancer cells during S phase leads to cellular senescence-like arrest within the same cell cycle. The mechanism of this arrest includes DNA strand breaking in S-phase cells, the collision of replication forks with the breaks, and the formation of difficult-to-repair DSBs [30]. Subsequently, persistent DDR results in proliferation arrest with a cellular senescence phenotype (Figure 1A). This mechanism is noteworthy because it utilizes extremely low concentrations of DNA-damaging agents (e.g., nanomolar concentrations of camptothecin was applied to the cells for 30-60 minutes) to induce cellular senescence [30]. Based on this mechanism, we developed an approach to remotely induce premature senescence in human cell cultures using short-term light irradiation. We employed the genetically encoded photosensitizers tKR and miniSOG and targeted them to chromatin to induce DNA lesions, which, in turn, induced difficult-to-repair DSBs, persistent DDR, and, subsequently, the development of the cellular senescence phenotype (Figure 1B). Briefly, the procedure used to induce cellular senescence includes the following steps: i) establishment of the cell line that transiently or stably expresses either tKR or miniSOG fused to core histone H2B to direct them to chromatin, ii) synchronization of the cells in S phase, and iii) light illumination of the cells (Figure 1B). In the current study, we generally used human HeLa Kyoto cell lines that stably express H2B-tKR or H2B-miniSOG, which were previously established by lentiviral transduction with corresponding constructs [17, 32]. We should mention that the expression levels of H2B-tKR (in contrast to H2B-miniSOG) fusion protein vary significantly across the population of stably transfected cells. This follows from a high cellular heterogeneity of H2B-tKR fusion protein fluorescence (data not shown). This observation is in agreement with the results of quantitative RT-PCR showing that the expression of H2B-miniSOG in the HeLa Kyoto cell line, which stably expresses this fusion protein, is approximately sevenfold higher than the expression of H2B-tKR in the corresponding cell line (data not shown). It was shown earlier that the fusion of tKR or miniSOG to core histone H2B effectively targets them to chromatin but does not induce cell killing or cell cycle alterations until the cells are illuminated with a specific wavelength of light [17, 19]. To synchronize the cells in S phase, we performed a double thymidine block, although one can use any other synchronization technique. Moreover, an asynchronous cell population can also be used; in this case, only the S-phase cells will senesce. To activate H2B-miniSOG or H2B-tKR, we illuminated the corresponding cell lines with blue light (465/95 nm, 65 mW/cm^2^) for 5 minutes or with green light (540/80 nm, 200 mW/cm^2^) for 15 minutes, respectively. As it will be shown below such illumination conditions were sufficient to induce cellular senescence but did not lead to cell killing.

### DNA damage induced by miniSOG and tKR

First, we analyzed whether genetically encoded photosensitizers targeted to chromatin could induce DNA strand breaks. Photosensitizers produce different ROS upon light-induced activation: O_2_^−^, hydrogen peroxides and hydroxyl radicals by the Type I photosensitization reaction and ^1^O_2_ by the Type II reaction. It is generally thought that these ROS can induce DNA damage. Indeed, it is quite well defined that hydrogen peroxide (H_2_O_2_) and hydroxyl (¯OH) stimulate DNA strand breaks, along with base and sugar oxidation [33]. It is much more complicated to determine the DNA-damaging effects of O_2_^−^ and ^1^O_2_. Although O_2_^−^ does not interact with undamaged DNA, it was shown that it could react with oxidatively generated DNA base radicals [34, 35]. Furthermore, O_2_^−^ can give rise to H_2_O_2_ and ¯OH radicals via a two-stage reaction [28]. ^1^O_2_ specifically reacts with guanines, thus producing 8-oxoguanines in DNA; however, the question of whether ^1^O_2_ can induce DNA strand breaks is still open [36, 37]. It should also be mentioned that the DNA-damaging effects of O_2_^−^ and ^1^O_2_ have been studied *in vitro* using free DNA in aqueous solutions. Therefore, it is questionable whether these ROS react with chromatin in living cells in a similar fashion. It is unclear what type of DNA damage can be induced by the genetically encoded photosensitizers, such as miniSOG and tKR. It had been only reported that the chromatin-targeted tKR could oxidize DNA bases [17, 27]. It is established that light-illumination of miniSOG leads to ^1^O_2_ production [19, 26], but the activation of tKR predominantly results in the formation of O_2_^−^; however, the possibility that tKR also produces ^1^O_2_ was not fully excluded [24, 25, 38]. Here, we investigated whether the chromatin-targeted miniSOG and tKR could induce DNA strand breaks upon activation by light illumination. For this purpose, we used the single-cell gel electrophoresis (SCGE) technique, also known as the “comet assay” [39]. The tail moment, the most meaningful parameter of the comet, which represents the tail length multiplied by the fraction of DNA in the tail, was chosen as a criterion for the degree of DNA breakage.

**Figure 2:**
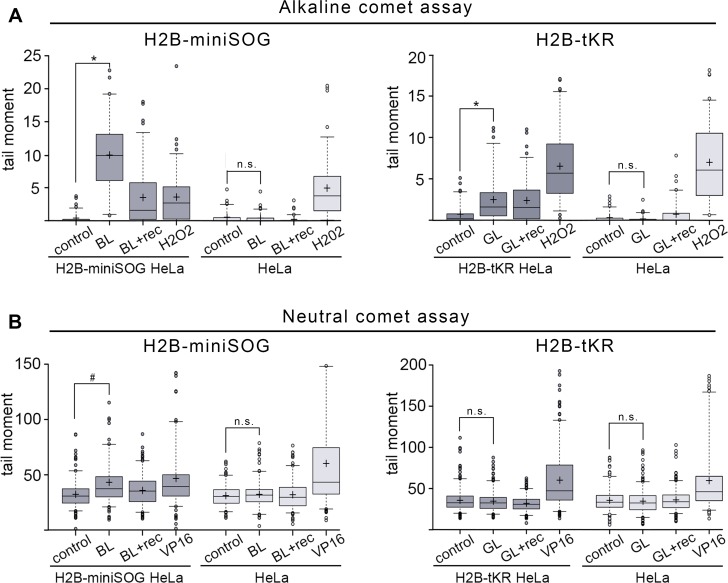
DNA damage induced by the activation of miniSOG and tKR targeted to chromatin (**A-B**) Asynchronous H2B-miniSOG expressing HeLa cells, along with their non-expressing counterparts, were either blue-light irradiated (“BL”; 465-495 nm, 65 mW/cm^2^, 5 min) or light irradiated and recovered for 30 min (“BL+rec”). Asynchronous H2B-tKR expressing HeLa cells, along with their non-expressing counterparts, were either green-light irradiated (“GL”; 540-580 nm, 200 mW/cm^2^, 15 min) or light irradiated and recovered for 30 min (“GL+rec”). Alkaline (**A**) and neutral (**B**) comet assays were performed. Non-illuminated cells were used (“control”) as a negative control and cells treated with H_2_O_2_ (“H2O2”; 200 μM, 1 hr) were used as a positive control in the alkaline comet assay (**A**), and cells treated with the topoisomerase II poison etoposide (“VP16”; 10 μg/ml, 1 hr) were used as a positive control in the neutral comet assay (**B**). Box plots show the tail moments. The boxed region represents the middle 50% of the tail moments, the horizontal lines represent the medians, and the black crosses indicate the means. *P < 0.0001 (two-tailed *t*-test, *n* > 70), ^#^*P* < 0.0001 (two-tailed *t*-test, n > 150), n.s. – not significant. The results of one of four experiments are shown.

First, we analyzed SSB generation in asynchronous HeLa cells expressing either H2B-miniSOG or H2B-tKR that were illuminated with the corresponding light source. We used an alkaline modification of the comet assay to perform this analysis. As a positive control for the presence of SSB, we used H_2_O_2_-treated cells. Blue- or green-light irradiation by itself did not induce any detectable DNA damage in control cells (Figure 2A). However, both chromatin-targeted miniSOG and tKR induced a significant number of SSBs (Figure 2A). As expected, miniSOG known to produce ^1^O_2_ [19, 26], had a much more pronounced DNA-damaging effect than tKR; it was comparable to the effects of high concentrations of H_2_O_2_ (200 μM, 1 h). However, this may also be due to several-fold higher level of expression of H2B-miniSOG fusion relative to H2B-tKR fusion (data not shown). It is interesting that although a significant portion of the SSBs induced by miniSOG were repaired within 30 minutes after illumination (Figure 2A, “BL+rec”), the tKR-induced lesions remained unresolved at this time point (Figure 2A, “GL+rec”). The mechanism of tKR-dependent SSB formation is elusive; the only possible (known) way for O_2_^−^ to induce DNA strand breaks is to be converted into H_2_O_2_ and ¯OH in a two-step reaction utilizing superoxide dismutase (SOD) and active metal ions [28]. However, the presence of SOD in the nuclei of the untreated cells is controversial – it was recently shown that Sod1 was only translocated from the cytoplasm to the nucleus only upon oxidative stress (treatment of cells with H_2_O_2_) [40]. It may be that tKR still generates a number of ^1^O_2_ that is responsible for SSB generation in this case.

We next analyzed DSB generation in asynchronous Hela cells expressing either H2B-miniSOG or H2B-tKR. For this purpose, we used the neutral comet assay; as a positive control for the presence of DSBs, we used cells that had been treated with the topoisomerase II poison etoposide (VP16; 10 μg/ml, 1 hr). Similar to SSBs, DSBs were not induced in response to illumination in HeLa cells that did not express the photosensitizers (Figure 2B). DSBs were only generated in HeLa cells expressing H2B-miniSOG that were illuminated with blue light (Figure 2B). Collectively, these results suggest that although both chromatin-targeted photosensitizers effectively stimulate SSB formation upon light irradiation, miniSOG can also produce DSBs.

### Light-induced cellular senescence

**Figure 3:**
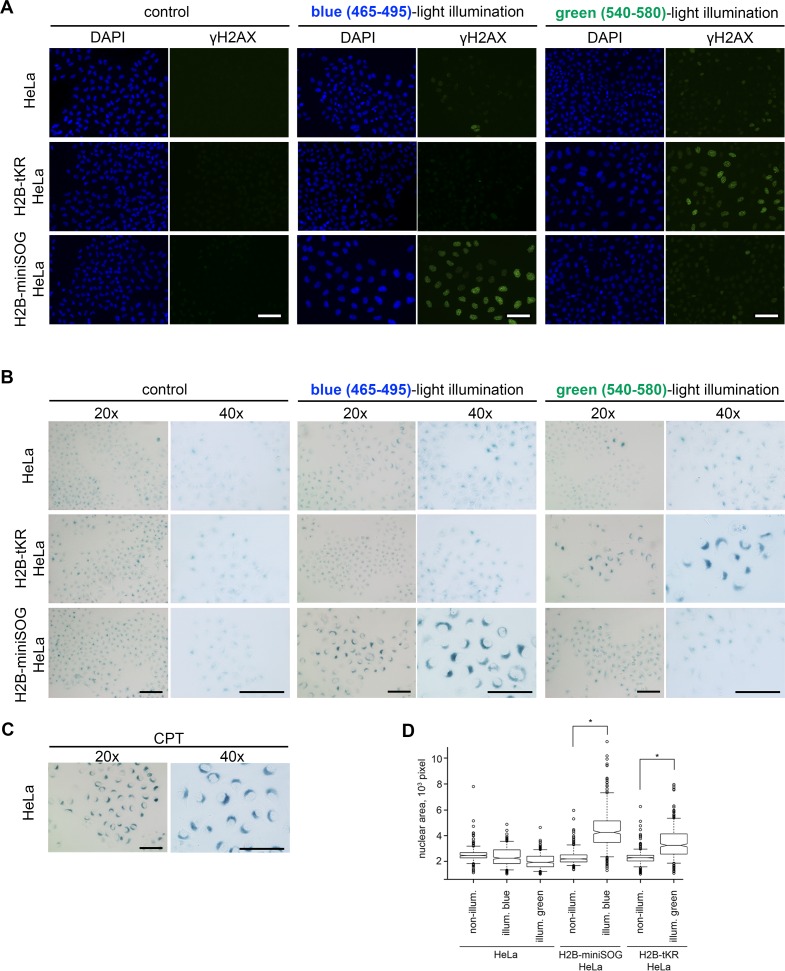
Activated genetically encoded photosensitizers can induce cellular senescence (**A-B**) The HeLa Kyoto cell line and its derivatives expressing either H2B-miniSOG or H2B-tKR were synchronized in S phase, illuminated with blue (465-495 nm, 65 mW/cm^2^, 5 min) or green (540-580 nm, 200 mW/cm^2^, 15 min) light, allowed to recover for 48 hr, and stained for γH2AX (**A**) or SA-β-gal (**B**). Control represents the cells that were synchronized and released for 48 hr (non-illuminated). The DNA was stained with DAPI in (**A**). Scale bar: 50 μm. (**C**) Senescent HeLa cells stained for SA-β-gal. Cellular senescence was induced by treatment of S-phase HeLa cells with a DNA topoisomerase I inhibitor camptothecin (1 μM, 1 h). (**D**) The HeLa Kyoto cell line and its derivatives expressing either H2B-miniSOG or H2B-tKR were synchronized in S phase, illuminated with corresponding light, allowed to recover for 48 hr, and stained with DAPI. Segmentation of cell nuclei was performed using CellProfiler. Boxplots show nuclear area in each case (*P=0.0001, two-tailed *t*-test).

**Figure 4:**
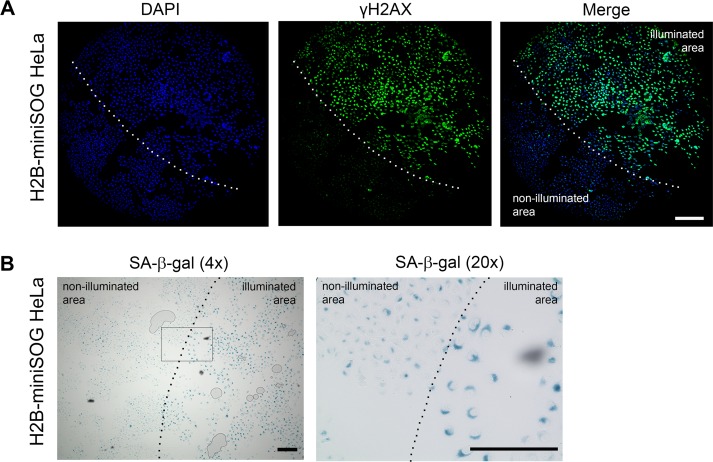
Locally activated H2B-miniSOG can induce cellular senescence (**A-B**) HeLa cells expressing H2B-miniSOG were synchronized in S phase, illuminated with blue (465-495 nm, 65 mW/cm^2^, 5 min) light, allowed to recover for 24 hr, and stained for γH2AX (**A**) or SA-β-gal (**B**). Only part of each specimen was illuminated. Dashed line shows the boundary between illuminated and non-illuminated parts of the specimens. Scale bars: 100 μm (**A**) and 80 μm (**B**).

Cellular senescence may be recognized by the manifestation of several typical signs, including cell and nuclear enlargement, senescence-associated β-galactosidase activity (SA-β-gal), the formation of senescence-associated heterochromatin foci (SAHF) and persistent DDR foci, senescence-associated secretory phenotype (SASP), increased expression of cyclin-dependent kinase inhibitors, etc. [41, 42]. The composition of these markers in a particular cellular senescence state greatly depends on the cell type and on the senescence-inducing stimulus. To test whether light irradiation of the chromatin-targeted photosensitizers induces cellular senescence, we first investigated DDR focus formation. For this purpose, the “parent” HeLa Kyoto cell line and its derivatives that stably express H2B-miniSOG or H2B-tKR were synchronized in S phase, light irradiated (465/95 and 540/80 nm), incubated for 48 hours, and immunostained with an antibody against γH2AX (phosphorylated at serine-139 variant histone H2AX), which is an ubiquitous DDR marker [43, 44]. It is evident that extensive DDR foci formation was only observed in the photosensitizer-expressing HeLa cells that had been illuminated with the relevant light (blue or green) (Figure 3A and 4A). It should be highlighted that γH2AX foci were not formed in the non-irradiated cells or in cells that were illuminated with an inappropriate light (blue light for tKR, and green light for miniSOG). This apparently means that the light-irradiation conditions (wavelength, power, and time) are not toxic to the cells by themselves. It is also worth noting that the cells exhibiting extensive γH2AX staining possessed enlarged nuclei, which is another senescence biomarker (Figure 3D).

To ascertain that the light irradiation-induced state does represent cellular senescence, we assayed the cells that were treated as described above for SA-β-gal activity, the most universal feature of cellular senescence [45]. As expected, only HeLa cells that expressed the photosensitizers and were irradiated with the appropriate light exhibited increased SA-β-gal activity (Figure 3B-C and 4B).

**Figure 5:**
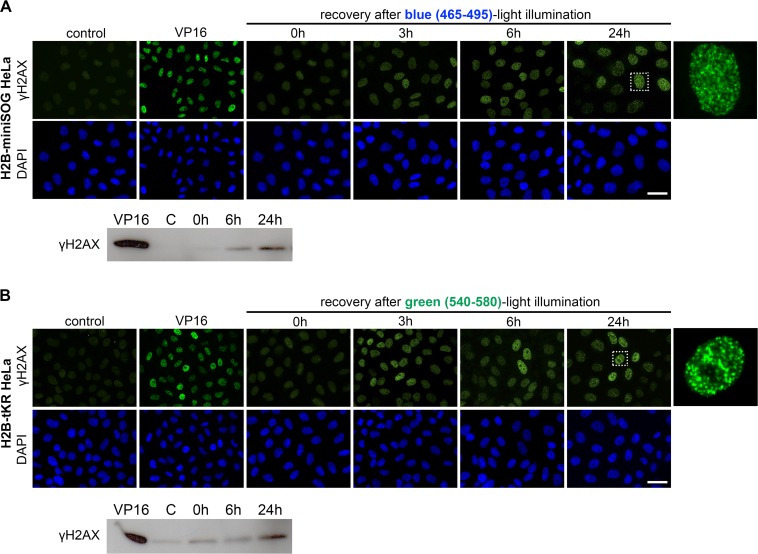
Temporal kinetics of the formation of the persistent DNA damage response foci induced by activation of miniSOG or tKR (**A-B**) HeLa cells that express H2B-miniSOG (**A**) or H2B-tKR (**B**) were synchronized in S phase, illuminated with blue (465-495 nm, 65 mW/cm^2^, 5 min) or green (540-580 nm, 200 mW/cm^2^, 15 min) light, allowed to recover for the indicated time intervals (0, 3, 6 and 24 hr). Histone γH2AX was analyzed by indirect immunofluorescence or WB. Negative control represents the cells that were synchronized but not light illuminated; positive control represents the cells treated with DNA topoisomerase II inhibitor etoposide (VP16; 10 μg/ml, 1 hr). The DNA was stained with DAPI. Scale bar: 20 μm.

To investigate the temporal kinetics of cellular senescence we assessed the presence of γH2AX foci in HeLa cells expressing either H2B-miniSOG or H2B-tKR that were light irradiated and recovered for different time periods (0, 3, 6 and 24 hours) (Figure 5A-B). We found that γH2AX foci were formed during the first hours, but not immediately after light-illumination, and did not disappear, thus, forming persistent DDR foci (Figure 5A-B). Western blot analysis of γH2AX fully confirmed the results obtained using indirect immunofluorescence (Figure 5A-B). Together, these observations support our model of delayed replication-dependent induction of difficult-to-repair DSBs [30].

**Figure 6:**
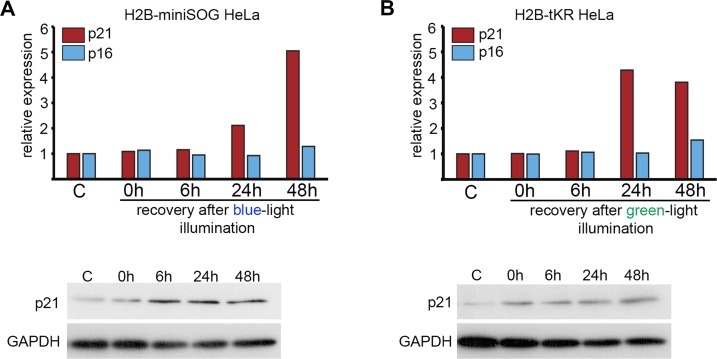
Analysis of the expression of p21 and p16 CDK inhibitors in HeLa cells expressing genetically encoded photosensitizers HeLa cells that express H2B-miniSOG or H2B-tKR were synchronized in S phase, illuminated with blue (465-495 nm, 65 mW/cm^2^, 5 min) or green (540-580 nm, 200 mW/cm^2^, 15 min) light, allowed to recover for the indicated time intervals (0, 6, 24 and 48 hr), and subjected to gene expression analysis using qRT-PCR and WB. Control (“C”) represents the non-illuminated cells. The expression of p21^CIP1^ and p16^INK4a^ was analyzed using EvaGreen-based qRT-PCR. The amplification levels of the cDNA were normalized to the level of the GAPDH cDNA. The results of one representative experiment are shown. WB was performed with an antibody against p21; GAPDH was used as the loading control.

It is well known that the senescence state is maintained by either p16^INK4A^- or p21^CIP1^-dependent signaling pathways [41]. In DNA damage-induced cellular senescence, the expression of p21^CIP1^ is usually increased. Using quantitative reverse transcription-PCR (qRT-PCR) and western blotting, we found that p21^CIP1^ but not p16^INK4A^ was upregulated in response to light irradiation of HeLa cells that express either H2B-miniSOG or H2B-tKR (Figure 6A-B). Interestingly, p21^CIP1^ expression was upregulated only after a pro-tracted recovery period (24 h) and not immediately after illumination (Figure 6A-B).

Finally, we decided to test whether the approach proposed is effectively compatible with a transient expression of these genetically encoded photo-sensitizers. For this purpose, we analyzed the induction of cellular senescence in HeLa cells that were transiently transfected with either H2B-miniSOG or H2B-tKR constructs, and then illuminated with an appropriate light. The results obtained clearly show that one can use transient transfection with genetically encoded photosensitizers to induce cellular senescence (Supplementary Figure 1).

In summary, we conclude that the HeLa cells that express H2B-fused photosensitizers acquire a senescence phenotype upon illumination with the appropriate light source in early S phase. This pheno-type is characterized by nuclear enlargement, the appearance of DDR foci, increased SA-β-gal activity, and the expression of the CDK inhibitor p21^CIP1^ (Figures 3 and 4). Interestingly, premature senescence was induced much more effectively in HeLa cells that expressed H2B-miniSOG. This is apparent from the fact that, in contrast to the H2B-miniSOG-expressing HeLa cells, not all of the H2B-tKR-expressing cells senesce in response to light illumination (Figure 2A and B). This difference may reflect the higher level of H2B-miniSOG expression and increased efficiency of miniSOG in inducing ROS production.

## CONCLUSIONS

Cellular senescence is one of the stress response pathways, in addition to apoptosis, autophagy, necrosis, etc. Although the contribution of cellular senescence to organismal ageing is still debatable [46], it is clear that cellular senescence has well-defined physiological functions [47, 48]. One of the most studied, physiologically relevant roles of senescence is cancer prevention [48]. The senescence phenotype can be induced *in vitro* by oncogene overexpression and/or DNA damage induction. Here, we suggest an approach to remotely activate the cellular senescence program. We used the genetically encoded photosensitizers tKR and miniSOG and targeted them to chromatin by fusing them to the core histone H2B to induce moderate levels of DNA damage using light (Figure 1). To induce senescence, cells expressing these modified photo-sensitizers should be synchronized in S phase and illuminated with an appropriate light source for a short time. The advantages of our approach are the following: i) the DNA damage is induced remotely without interfering with the cell culture; ii) the resulting DNA damage is adjustable by varying the light irradiation, time, and/or power; and iii) all types of fluorescent microscopes are appropriate for this method. The most useful feature of the approach is the possibility to remotely induce cellular senescence in a cell population or even in individual cells using light-illumination. This is applicable in studies of cellular senescence using fast-evolving lab-on-a-chip technologies [49], specifically, to investigate cell-to-cell communication or the cellular secretome.

Here, we also shed light on the question of whether genetically encoded photosensitizers can induce DNA damage, particularly DNA strand breaking. We showed that both chromatin-targeted tKR and miniSOG induced SSBs upon light activation; however, only miniSOG was able to induce DSBs (Figure 4). This difference may be related to the fact that miniSOG produces highly active ^1^O_2_, whereas tKR produces O_2_^−^ [19, 25]. Nevertheless, this is the first direct demonstration of DNA strand breaks produced by genetically encoded photosensitizers localized in the cell nucleus, which allows them to be used for DNA damage studies. These findings can stimulate the development of new strategies for using photosensitizers in translational applications. It is also worth noting that we could investigate the effects of different ROS - ^1^O_2_ and O_2_^−^ - on DNA using tKR and miniSOG targeted to chromatin. Nevertheless, the question of whether and how different ROS induce DNA damage is far from being fully understood.

## METHODS

### Cell culture, synchronization and transfection

HeLa cell line and HeLa Kyoto cell lines that stably express either H2B-tKR or H2B-miniSOG and their non-expressing counterparts were used. The cells were cultured in DMEM (PanEco) supplemented with 10% fetal bovine serum (FBS; HyClone/GE Healthcare), 10 U/ml penicillin (PanEco) and 10 μg/ml streptomycin (PanEco). The cells were grown at 37°C and 5% CO_2_ in a conventional humidified CO_2_ incubator. For synchronization by double-thymidine block, 30% confluent cells were incubated with 2 mM thymidine for 16 h, released from the block for 9 h, and then incubated with thymidine for an additional 16 h. To release the cells from thymidine, they were washed twice with phosphate-buffered saline (PBS) and incubated in fresh culture medium.

HeLa cells were transfected with 2 μg of either H2B-miniSOG or H2B-tKR construct using Xfect transfection reagent (Clontech) following the manufac-turer's instructions. After 24 h, cells were illuminated to induce cellular senescence. The efficiency of trans-fection was estimated by fluorescence of genetically encoded photosensitizers transfected; usually more than 80% of cells were transfected.

### Cell illumination

An inverted Nikon Eclipse Ti-E fluorescence microscope equipped with a Nikon Intensilight C-HGFI light source and a Nikon Plan Fluor 4x/0.13 objective was used to illuminate the cells. A standard filter set was used to acquire the relevant fluorescence signals and to illuminate the cells. The FITC filter set was used for blue-light illumination (excitation band pass (BP) 465/95 nm, emission BP 515/55 nm), and the Tx Red filter set was used for green-light illumination (excitation BP 540/80 nm, emission BP 600/60 nm). A Laser Power Meter LP1 (Sanwa) was used to measure the total power of the excitation light. The light power density (W/cm^2^) was estimated by dividing the total power by the area of the illuminated region. The cells stably expressing H2B-miniSOG were illuminated for 5 min with blue light (65 mW/cm^2^ light power density), and the H2B-tKR expressing cells were illuminated for 15 min with green light (200 mW/cm^2^ light power density).

### Indirect immunofluorescence

Cells were grown on microscope slides, illuminated, fixed, and permeabilized in CSK buffer (10 mM PIPES, pH 7.0, 100 mM NaCl, 1.5 mM MgCl_2_, and 300 mM sucrose) supplemented with 1% PFA and 2.5% Triton X-100 for 15 min at room temperature. The fixed cells were washed three times for 5 min in PBS. After washing, the cells were preincubated with 1% BSA in PBS for 30 min and then incubated with a primary antibody against γH2AX (Upstate/Millipore, #05-636) in PBS supplemented with 1% BSA for 1 h at room temperature. The cells were washed three times for 5 min with PBS. To visualize the primary antibodies, the samples were incubated with Alexa Fluor 488- or Alexa Fluor 594-conjugated secondary antibodies (Molecular Probes/Life Technologies). The DNA was counter-stained with the fluorescent dye 4,6-diamino-2-phenylindole (DAPI) in PBS for 5 min at room temperature. After washing in PBS and distilled water, the samples were mounted using Dako fluorescent mounting medium (Life Technologies). The immuno-stained samples were analyzed using a Zeiss AxioScope A.1 fluorescence microscope (objective: Zeiss N-Achroplan 40×/0.65; camera: Zeiss AxioCam MRm; acquisition software: Zeiss AxioVision Rel. 4.8.2; Jena, Germany). The images were processed using ImageJ software (version 1.44) and Adobe Photoshop CS6.

### SA-β-galactosidase assay

The cells were fixed in 2% formaldehyde and 0.2% glutaraldehyde in PBS for 5 min at room temperature. Then, the cells were washed three times in PBS for 5 min and incubated in staining solution (1 mg/ml 5-bromo-4-chloro-3-indolyl β-D-galactopyranoside (X-gal; Sigma-Aldrich), 40 mM citric acid/sodium phosphate buffer, pH 6.0, 150 mM NaCl, 2 mM MgCl_2_, 5 mM K_3_Fe(CN)_6_, and 5 mM K_4_Fe(CN)_6_) for 18 h at 37°C. Then, the samples were washed in PBS for 5 min and fixed with methanol for 5 min at room temperature.

### Neutral comet assay

After illumination, the cells were immersed in Versen solution and incubated at 37°C for 5 min. The cell suspension was then mixed in a 1:1 ratio with a Trevigen LMAgarose (#4250-050-02) at 37°C. The mixture was pipetted onto comet slides (Trevigen, #3950-300-02) that had been precoated with a 1% normal melting point agarose base layer. The drop containing the cells was covered with a glass cover slip and incubated at 4°C for 5 min. The cover slips were removed, and the slides were then immersed in lysis solution (30 mM EDTA, 0.5% SDS, and 10 mM Tris-HCl, pH 8.0, supplemented with 200 mg/ml proteinase K (Sigma-Aldrich)) at 37°C for 1 h. After lysis, the slides were washed three times for 5 min in PBS and incubated in 1x TBE for 20 min at 4°C. Electrophoresis was performed in a Trevigen electrophoresis system (#4250-050-ES) for 20 min at 4°C and 1 V/cm in 1x TBE. After washing with PBS, the slides were stained with a 1:3000 dilution of SYBR Green (Thermo Scientific, #S7563). The comets were visualized at 4x magnification using an inverted Nikon Eclipse Ti-E fluorescence microscope equipped with a Nikon Intensilight C-HGFI light source (objective: Nikon Plan Fluor 4x/0.13; camera: DS-Qi2). The images of the comets were analyzed with the CometScore software. Statistical analysis was performed using IBM SPSS Statistics 20.

### Alkaline comet assay

The preparation of the samples and slides was similar to the neutral comet assay. The slides were immersed in lysis solution (10 mM Tris-base, 2.5 M NaCl, 100 mM EDTA, 1% Triton X-100, and 10% DMSO, pH 10.0) for 1 h at 4°C in the dark. Then, the slides were immersed in unwinding buffer (300 mM NaOH and 1 mM EDTA, pH 13.0) for 20 min at 4°C. The slides were subjected to electrophoresis in a Trevigen electrophoresis system (#4250-050-ES) for 20 min at 4°C and 0.7 V/cm in unwinding buffer. After electrophoresis, the slides were neutralized with 500 mM Tris-HCl, pH 8.0, and stained with a 1:3000 dilution of SYBR Green (Thermo Scientific, #S7563). The comets were visualized at 4x magnification using an inverted Nikon Eclipse Ti-E fluorescence microscope equipped with a Nikon Intensilight C-HGFI light source (objective: Nikon Plan Fluor 4x/0.13; camera: DS-Qi2). The images of the comets were analyzed with the CometScore software. Statistical analysis was performed using IBM SPSS Statistics 20.

### RNA isolation and reverse transcription quantitative PCR

The total RNA was extracted from the cells using TRIzol reagent (Life Technologies), and cDNA synthesis was performed at 42°C for 1 h using 0.5 μg of the total RNA as a template, 0.4 μg of random hexamer primers and 200 U of reverse transcriptase (Fermentas) in the presence of 20 U of ribonuclease inhibitor (Fermentas). The obtained cDNAs were analyzed by quantitative polymerase chain reaction (PCR) using the CFX96 real-time PCR detection system (Bio-Rad Laboratories). Each reaction contained 50 mM Tris-HCl, pH 8.6, 50 mM KCl, 1.5 mM MgCl_2_, 0.1% Tween-20, 0.5 μM each primer, 0.2 mM dNTPs, 0.6 μM EvaGreen (Biotium), 0.75 U of Hot Start Taq Polymerase (Sibenzyme) and 50 ng of cDNAs. The PCR cycling conditions were as follows: initial denaturation for 5 min at 94°C, 40 cycles of 15 s at 94°C, 30 s at 65°C and 15 s at 72°C. The gene-specific primer pairs were: GAPDH - aaactgtggcgtgatggc and cagtggggacacggaagg; p16 – acaactgcccccgccacaac and acagtgaaaaggcagaagcggtg; p21 – aaggcagggggaaggtgggg and gggggagggacagcagcaga; H2B-tKR – cagccaccacacctacgag and tgaagccgatgaaggccag; H2B-miniSOG – gctttgtgattaccgatccgc and attttctgcacggtggcttg.

### Whole-cell extracts preparation and immunoblotting

HeLa cells were lysed by incubation in RIPA buffer (150 mM NaCl, 1% Triton X-100, 0.5% sodium deoxycholate, 0.1% SDS, 50 mM Tris-HCl (pH 8.0), 1 mM dithiothreitol, and 1 mM PMSF) supplemented with Protease Inhibitor Cocktail (Sigma-Aldrich) and Phosphatase Inhibitor Cocktail 2 (Sigma-Aldrich) for 30 min on ice. Next, the cell extracts were sonicated with a VirSonic 100 ultrasonic cell disrupter and stored at −70°C. The protein concentration was measured by the Bradford assay. Aliquots of each sample were separated by SDS–PAGE and blotted onto PVDF membranes (Amersham/GE Healthcare). The membranes were blocked for 1 hr in 2% ECL Advance blocking reagent (GE Healthcare) in PBS containing 0.1% Tween 20 (PBS-T) followed by overnight incubation with a primary antibody diluted in PBS-T containing 2% blocking reagent. After three washes with PBS-T, the membranes were incubated for 1 hr with the secondary antibodies (horseradish peroxidase-conjugated anti-rabbit or anti-mouse IgG) in PBS-T containing 2% blocking agent. The immunoblots were visualised using a Pierce ECL plus western blotting substrate.

## SUPPLEMENTARY MATERIAL


