# Supplementary material for: Inducing cellular senescence in vitro by using genetically encoded photosensitizers
Source: Aging (Albany NY). 2016 Oct 14;8(10):2449–61. doi: 10.18632/aging.101065 (PMC5115900; doi:10.18632/aging.101065)
Supplement: Supplementary file 1 [file aging-08-2449-s001.pdf]

SUPPLEMENTARY MATERIAL

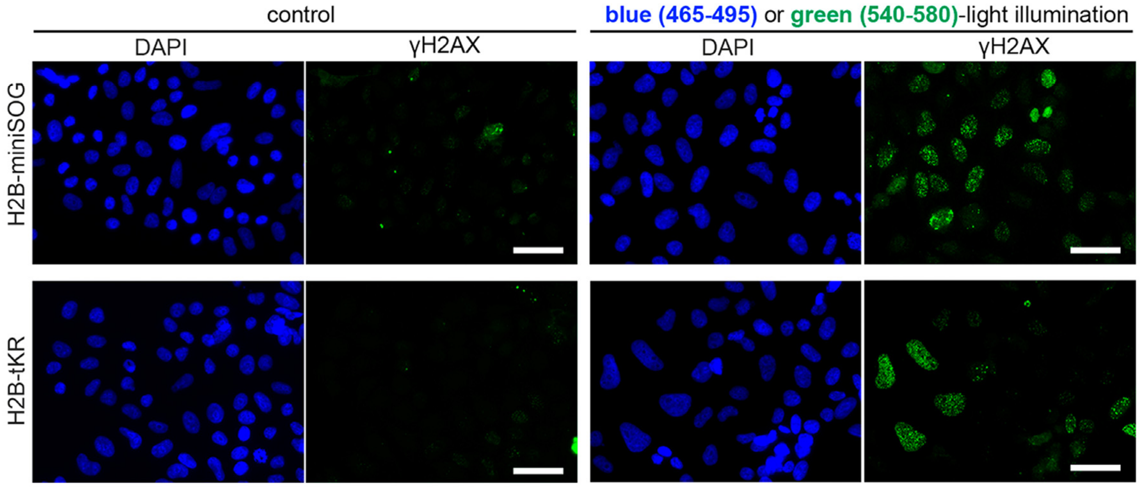

Figure S1. Human HeLa cells were transiently transfected either with H2B-miniSOG or H2B-tKR, synchronized in S phase, illuminated with an appropriate light, allowed to recover for 48 hours, and immunostained for γH2AX. Controls represent the cells that were transfected, synchronized and released for 48 hr (non-illuminated). The DNA was stained with DAPI. Scale bar: 30 μm.
